# Deep Learning Algorithm for Management of Diabetes Mellitus via Electrocardiogram-Based Glycated Hemoglobin (ECG-HbA1c): A Retrospective Cohort Study

**DOI:** 10.3390/jpm11080725

**Published:** 2021-07-27

**Authors:** Chin-Sheng Lin, Yung-Tsai Lee, Wen-Hui Fang, Yu-Sheng Lou, Feng-Chih Kuo, Chia-Cheng Lee, Chin Lin

**Affiliations:** 1 Division of Cardiology, Department of Internal Medicine, Tri-Service General Hospital, National Defense Medical Center, No 325, Section 2, Cheng-Kung Rd., Neihu, Taipei 114, Taiwan; littlelincs@gmail.com; 2 Division of Cardiovascular Surgery, Cheng Hsin Rehabilitation and Medical Center, No 45, Cheng Hsin St., Beitou, Taipei 112, Taiwan; andrewytlee.cvs@gmail.com; 3 Department of Family and Community Medicine, Department of Internal Medicine, Tri-Service General Hospital, National Defense Medical Center, No 325, Section 2, Cheng-Kung Rd., Neihu, Taipei 114, Taiwan; rumaf.fang@gmail.com; 4 Graduate Institute of Life Sciences, National Defense Medical Center, No.161, Section 6, Min-Chun E. Rd., Neihu, Taipei 114, Taiwan; chaos53438@gmail.com; 5 School of Public Health, National Defense Medical Center, No.161, Section 6, Min-Chun E. Rd., Neihu, Taipei 114, Taiwan; 6 Division of Endocrinology and Metabolism, Department of Internal Medicine, Tri-Service General Hospital, National Defense Medical Center, No 325, Section 2, Cheng-Kung Rd., Neihu, Taipei 114, Taiwan; shoummie@hotmail.com; 7 Department of Medical Informatics, Tri-Service General Hospital, National Defense Medical Center, No 325, Section 2, Cheng-Kung Rd., Neihu, Taipei 114, Taiwan; lcgnet@gmail.com; 8 Division of Colorectal Surgery, Department of Surgery, Tri-Service General Hospital, National Defense Medical Center, No 325, Section 2, Cheng-Kung Rd., Neihu, Taipei 114, Taiwan; 9 Medical Technology Education Center, School of Medicine, National Defense Medical Center, No.161, Section 6, Min-Chun E. Rd., Neihu, Taipei 114, Taiwan

**Keywords:** artificial intelligence, electrocardiogram, deep learning, glycated hemoglobin, diabetes mellitus

## Abstract

Background: glycated hemoglobin (HbA1c) provides information on diabetes mellitus (DM) management. Electrocardiography (ECG) is a noninvasive test of cardiac activity that has been determined to be related to DM and its complications. This study developed a deep learning model (DLM) to estimate HbA1c via ECG. Methods: there were 104,823 ECGs with corresponding HbA1c or fasting glucose which were utilized to train a DLM for calculating ECG-HbA1c. Next, 1539 cases from outpatient departments and health examination centers provided 2190 ECGs for initial validation, and another 3293 cases with their first ECGs were employed to analyze its contributions to DM management. The primary analysis was used to distinguish patients with and without mild to severe DM, and the secondary analysis was to explore the predictive value of ECG-HbA1c for future complications, which included all-cause mortality, new-onset chronic kidney disease (CKD), and new-onset heart failure (HF). Results: we used a gender/age-matching strategy to train a DLM to achieve the best AUCs of 0.8255 with a sensitivity of 71.9% and specificity of 77.7% in a follow-up cohort with correlation of 0.496 and mean absolute errors of 1.230. The stratified analysis shows that DM presented in patients with fewer comorbidities was significantly more likely to be detected by ECG-HbA1c. Patients with higher ECG-HbA1c under the same Lab-HbA1c exhibited worse physical conditions. Of interest, ECG-HbA1c may contribute to the mortality (gender/age adjusted hazard ratio (HR): 1.53, 95% conference interval (CI): 1.08–2.17), new-onset CKD (HR: 1.56, 95% CI: 1.30–1.87), and new-onset HF (HR: 1.51, 95% CI: 1.13–2.01) independently of Lab-HbA1c. An additional impact of ECG-HbA1c on the risk of all-cause mortality (C-index: 0.831 to 0.835, *p* < 0.05), new-onset CKD (C-index: 0.735 to 0.745, *p* < 0.01), and new-onset HF (C-index: 0.793 to 0.796, *p* < 0.05) were observed in full adjustment models. Conclusion: the ECG-HbA1c could be considered as a novel biomarker for screening DM and predicting the progression of DM and its complications.

## 1. Introduction

Diabetes mellitus (DM) is a critical public health issue, as this disease may affect 463 million people worldwide and will increase by 25% by 2030 and by 51% by 2045 [1]. Early detection of DM improves the quality of care, contributing to fewer complications, improved survival, and higher quality of life for patients [2]. The World Health Organization guidance proposed that a glycated hemoglobin (HbA1c) value of 6.5% is the cut-off point for diagnosing DM [3]. Although this invasive blood test may accurately identify potential DM without requiring fasting, it is limited by many conditions, causing it to be unpopular as a large-scale screening test. At present, there are various risk-scoring systems to use a set of noninvasive indicators for screening DM [4,5,6,7]. However, for these indicators, the area under the receiver operating characteristic curve (AUC) ranged from 0.72 to 0.81 in external validations [7]. Developing a more accurate noninvasive DM screening marker may reduce the burden of major complications of DM, including diabetic retinopathy (DR), diabetic neuropathy (DN), chronic kidney disease (CKD) and, particularly, cardiovascular diseases (CVDs) [8,9], which contribute to approximately 70% of DM-related deaths and significantly increase the medical costs of diabetic patients [10]. The American Diabetes Association guidelines have therefore recommended that healthcare systems should conduct regular assessments and management of complications in diabetic patients [11]. HbA1c is not only useful for DM screening, but is also employed to predict DR [12], DN [13], and CKD [14]. The increase in CVD risk with rising HbA1c levels starts even without DM [15]. However, HbA1c is not a regular laboratory test, which results in a large number of missing values [16], leading to difficulties when applying a screening system based on electronic medical records.

Currently, an increasing number of studies describe the use of unstructured data in the medical field [17]. For example, coronary artery calcium can be used to enhance the prediction of CVD risk [18], and another study extracted additional signals from free-text medical records on risk stratification [19]. In the past, many studies have attempted to obtain more information about the prognosis or disease diagnosis from electrocardiograms (ECG), and have successively helped to clarify the relationship between the ECG and the prognosis, but this method has not become popular because it is difficult to judge the waveform and requires other mathematical analyses. The current revolution in artificial intelligence (AI) based on deep learning models (DLMs) is a data-driven technique to learn useful features in an automated fashion [20], which is powerful for detecting myocardial infarction [21], digoxin toxicity [22], arrhythmia [23,24], hyperkalemia [25,26], left ventricular dysfunction [27,28], mitral regurgitation [29], aortic stenosis [30], and hypoglycemic events [31]. Therefore, we attempted to employ DLM to apply ECG to DM management, which may combine unstructured data to identify additional information.

DLM has also been shown to extract features that are unrecognizable to humans, such as sex and age [32]. Interestingly, patients with higher estimated ECG age, even with the same chronological age, usually have characteristics of hypertension, CAD, or a low ejection fraction [32]. We hypothesize that ECG may represent a novel biomarker for screening DM and predicting its progression. DLM may extract underlying factors when using ECG to estimate a DM progression index, such as estimating age via ECG. Because the HbA1c may be the most important factor in DM screening [3] and progression [33], we investigated the feasibility of estimating HbA1c by ECG. This study aimed to train a series of DLMs using ECG to predict HbA1c, and the ECG based HbA1c (ECG-HbA1c) was decided by the DLM performance comparison. We quantified its performance on DM screening, and the underlying characteristic differences in different ECG-HbA1c under the same laboratory-based HbA1c were also analyzed. Finally, we also attempted to use ECG-HbA1c to improve the predictive accuracy of all-cause mortality, new-onset CKD, and new-onset heart failure (HF) to validate the utility of this novel biomarker.

## 2. Materials and Methods

### 2.1. Data Source and Population

The electronic medical records of our hospital included digital ECG signals, and the records from 1 January 2012 to 31 December 2019 were available. ECGs were collected using a Philips 12-lead ECG machine (PH080A) with a 500-Hz sampling frequency and 10 s in each lead. The quantitative measurements and findings within the final ECG clinical reports were extracted to identify 31 diagnostic pattern classes and 8 continuous ECG measurements. The 8 ECG measurements included heart rate, PR interval, QRS duration, QT interval, correct QT interval, P wave axis, RS wave axis, and T wave axis. Data for these variables were 93–100% complete, and missing values were imputed using multiple imputations [34]. Patterns included abnormal T wave, atrial fibrillation, atrial flutter, atrial premature complex, complete AV block, complete left bundle branch block, complete right bundle branch block, first degree AV block, incomplete left bundle branch block, incomplete right bundle branch block, ischemia/infarction, junctional rhythm, left anterior fascicular block, left atrial enlargement, left axis deviation, left posterior fascicular block, left ventricular hypertrophy, low QRS voltage, pacemaker rhythm, prolonged QT interval, right atrial enlargement, right ventricular hypertrophy, second degree AV block, sinus bradycardia, sinus pause, sinus rhythm, sinus tachycardia, supraventricular tachycardia, ventricular premature complex, ventricular tachycardia, and Wolff–Parkinson–White syndrome. The 31 clinical diagnosis patterns were parsed from the structured findings statements on the basis of the key phrases that are standard within the Philips system. These features were used to train an extreme gradient boosting (XGB) model and elastic net, and the DLM was trained via raw ECG traces.

In this study, we used the HbA1c value and measured the method of ion-exchange through high performance liquid chromatography (HPLC) via running on the instrument of HLC-723G11. The ion-exchange HPLC method is certificated by the National glycohemoglobin standardization program (NGSP) as the HbA1c test with traceability to the Diabetes control and complications trial (DCCT) reference assay. The other laboratory-testing histories were collected from our electronic medical records. The diagnosis of DM was made by the following criteria [35]: (1) 6.5% or higher on two separate HbA1c tests; (2) 126 mg/dL or higher on two separate GLU tests; (3) 200 mg/dL or higher after two hours in oral glucose tolerance test. Patients meeting any of the above criteria before the date of ECG were identified. Moreover, patients with a HbA1c of ≥8.0% were defined as a severe DM at the date of ECG. In this study, we classified patients with prediabetes as a non-DM group in following analyses, which were defined with the following criteria: (1) 5.7% or higher on two separate HbA1c tests; (2) 100 mg/dL or higher on two separate GLU tests; (3) 140 mg/dL or higher after two hours in an oral glucose tolerance test.

**Figure 1:**
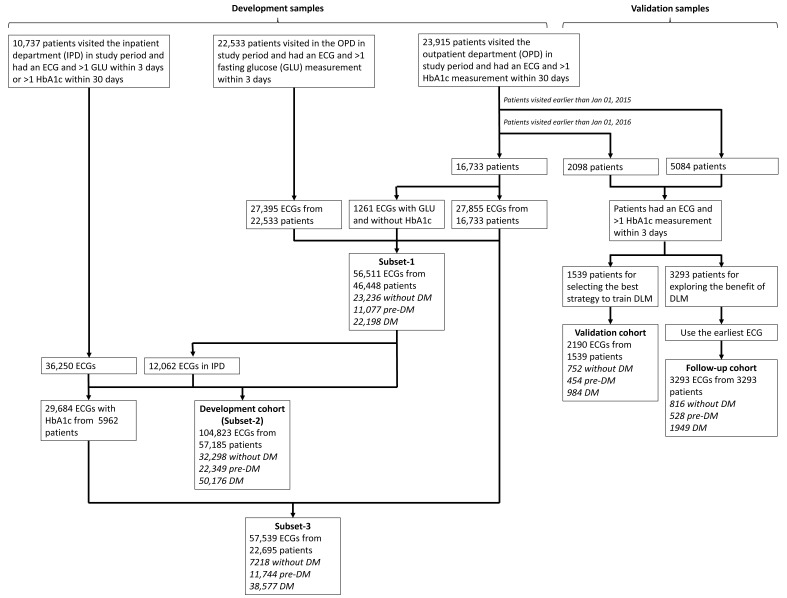
The summary of study design in this study. The process of development, validation, and follow-up cohorts with each electrocardiogram (ECG) labeling of HbA1c was indicated. The patients in validation and follow-up cohorts were totally different from development cohort. The development cohort included three subsets (subset-1: outpatient department samples; subset-2: full samples; subset-3: samples with corresponding HbA1c). Abbreviations: DM, diabetes mellitus.

Figure 1 shows the generation of study cohorts. There were 23,195 patients visiting the outpatient department (OPD) in the study period with more than 1 ECG and HbA1c pair within 30 days. Among them, there were 5084 patients visiting the OPD earlier than 1 January 2015, and 2098 patients visiting the OPD from 1 January 2015 to 31 December 2015. Only patients that had an ECG and >1 HbA1c measurement within 3 days were included, leading to the remaining 3293 patients and 1539 patients before 1 January 2015 and between 1 January 2015 and 31 December 2015, respectively. There were 2190 ECGs from 1539 people in the validation cohort for initially validating the DLMs. As for the 3293 patients, only the earliest ECG was applied to generate a follow-up cohort with 3293 ECGs, which were used for the accuracy test of DLM and the evaluation of the meaning of prediction error. In the validation cohort, 752 (34.3%) patients had no history of DM, 454 (20.7%) patients had the history of prediabetes with the duration of 1.19 ± 1.83 years, and 984 (44.9%) patients had the history of DM with the duration of 4.84 ± 3.83 years, while the follow-up cohort consisted of 816 (24.8%) patients without DM, 528 (16.0%) patients with the history of prediabetes and the duration of 1.75 ± 2.36 years, and 1949 (59.2%) patients with the history of DM and the duration of 4.69 ± 3.77 years. Based on this sample size for following the outcomes with hypothetical incidences of 1%/5%, the statistical powers achieved 65.6%/>99.9% using the following settings: a significance level of 0.05, a ratio of two groups was equal to 1, and a minimum detectable relative risk of 2. We selected the earliest data as the follow-up cohort for maximizing the following time of DM related outcomes. There was no overlap among the cohorts.

We used a series of methods to collect more samples for developing DLMs. The remaining 16,733 patients first visited after 1 January 2016 had 27,855 ECGs with corresponding HbA1c in the OPD. For further increasing the data volume, we included ECGs without corresponding HbA1c but with corresponding fasting glucose (GLU) within 3 days. A previous study developed an equation for estimating average GLU as follows: 28.7 × HbA1C—46.7 [36], and we used the inverse function to calculate the estimated HbA1c. This method increased to 1261 ECGs with corresponding GLU and without HbA1c from 16,733 patients with more than 1 ECG and GLU pair. Further, 27,395 ECGs with estimated HbA1c from the other 22,533 patients in study period were collected. Therefore, a total of 56,511 ECGs from 46,448 patients were used to construct subset-1 with only OPD data included. To further augment the development samples, 10,737 patients who visited the inpatient department (IPD) were included in the study period, with 36,250 ECGs and corresponding HbA1c within 30 days or estimated HbA1c within 3 days. There were 12,062 IPD ECGs from the 46,448 patients in subset-1 using the same criteria, and there were 104,823 ECGs from 57,185 patients in the subset-2. We further excluded the ECGs without corresponding HbA1c to construct subset-3 with the remaining 57,539 ECGs from 22,695 patients. We defined the subset-2 as the major development cohort. There were 32,298 (30.8%) patients without DM, 22,695 (21.3%) patients with the history of prediabetes and the duration of 0.88 ± 1.91 years, and 50,176 (47.9%) patients with history of DM and the duration of 4.33 ± 4.07 years.

### 2.2. Observational Variables

In addition to glucose profile, we also collected the relevant blood laboratory values in the OPD, including electrolytes, liver and renal function profiles, albumin (Alb), c-reactive protein (CRP), complete blood cell count, and lipid profiles. The nearest laboratory test was obtained within 3 days before and after enrollment. The missing data were imputed using multiple imputations in multivariable analysis [34].

The complications of this study in the follow-up cohort were all-cause mortality, new-onset CKD, and new-onset HF. For the mortality data, the survival time was calculated with reference to the date of ECG. Patient status (dead/alive) was defined through electronic medical records, which were updated by each hospital activity. Moreover, data for alive visits were censored at the patient’s last known hospital alive encounter to limit bias from incomplete records. The end of follow-up in this study was 31 December 2019. Patients without revisits to our hospital were excluded, and there were 3288 (99.8%) at risk samples for mortality analysis.

The new-onset CKD event was defined as at least 2 records of estimated glomerular filtration rate (eGFR) ≤60 mL/min or markers of kidney damage (albumin to creatinine ratio ≥30 mg/g or positive urine strip test) after the index date. Patients meeting any of the above criteria before the date of ECG were excluded and defined as having CKD history, and the number of at risk patients was 2426. The HF was defined by the quantitative ejection fraction recorded at the acquisition in the Philips image system^®^. The ejection fraction is routinely acquired by experienced cardiologist or technicians using a standardized method. An ejection fraction of ≤35% was defined as HF in this study, and the history of HF and at risk patients followed the above rules. There were 3031 at risk patients to follow up on the new-onset HF.

The other disease histories were based on the corresponding International Classification of Diseases, Ninth Revision and Tenth Revision (ICD-9 and ICD-10, respectively) as follows: hypertension (HTN, ICD-9 codes 401.x to 404.x and ICD-10 codes I10.x to I16.x), hyperlipidemia (HLP, ICD-9 codes 272.x and ICD-10 codes E78.x), stroke (STK, ICD-9 codes 430.x to 438.x and ICD-10 codes I60.x to I63.x), coronary artery disease (CAD, ICD-9 codes 410.x to 414.x, and 429.2, and ICD-10 codes I20.x to I25.x), atrial fibrillation (AF, ICD-9 codes 427.31 and ICD-10 codes I48.x), and chronic obstructive pulmonary disease (COPD, ICD-9 codes 490.x to 496.x and ICD-10 codes J44.9).

### 2.3. Implementation of the Deep Learning Model

**Figure 2:**
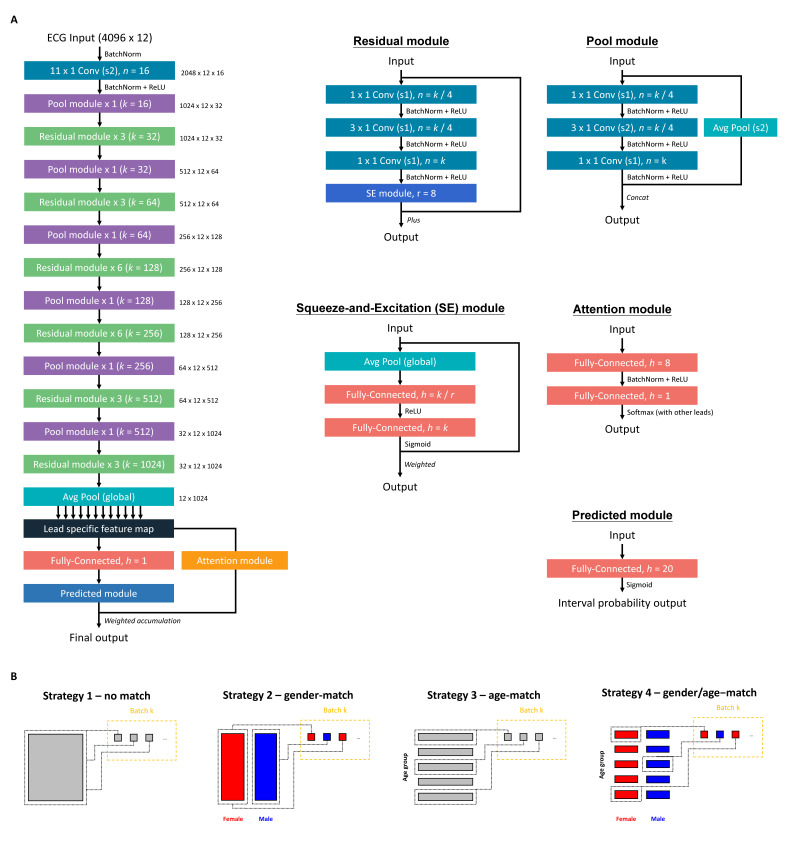
The implementation of our deep learning model. (**A**) The model architectures of the deep learning model for analyzing ECG. (**B**) Four training strategies were based on different sampling processes. The matching strategy was to split the sample to multiple blocks based on different conditions. The batch samples were sampled from each block with the same probability.

The DLM architecture with an attention mechanism was used to estimate HbA1c, which was based on our previous study [21,22,26,37]. Figure 2A shows the architecture of our DLM. Each ECG was recorded as a standard 12 leads consisting of 5000 number sequences, and a 5000 × 12 matrix was generated based on these sequences. An input format of this architecture is a 4096 × 12 matrix. We randomly cropped a length of 4096 sequences as input during the training process. For the inference stage, 2 overlapping lengths of 4096 sequences at the start and the end were used to generate predictions that were averaged as the final prediction.

We defined a “residual module” as a neural combination with a constant *k*, as follows: (1) a 1 × 1 convolution layer with *k*/4 filters to reduce the dimensions of the data, (2) a batch normalization layer to normalization, (3) a rectified linear unit (ReLU) layer for non-linearization, (4) a 3 × 1 convolution layer with *k*/4 filters to extract features, (5) a batch normalization layer for normalization, (6) a ReLU layer for non-linearization, and (6) a 3 × 1 convolution layer with 4K filters to extract features, (7) a 1 × 1 convolution layer with *k* filters to restore feature shape, (8) a batch normalization layer for normalization, (9) a ReLU layer for non-linearization, and (10) a squeeze-and-excitation (SE) module for weighting features. The SE module was defined as follows: (1) an average global pooling layer, (2) a fully-connected layer with *k*/*r* neurons, and (3) a fully-connected layer with *k* neurons. The constant *r* was set at 8 in all experiments. The residual module was ended by a shortcut connection, resulting in direct connections of each layer with all subsequent layers.

The residual module cannot be concatenated when the size of feature maps changes. Thus, a “pool module” was used to concatenate each residual module for down-sampling in our architecture. This module included similar concatenated layers with residual modules, but the stride of the 3 × 1 convolution layer was changed to 2 × 1. An average pooling layer with a 2 × 1 kernel size and stride was used for down-sampling. We used the concatenated function to integrate them.

The input data were passed through a batch normalization layer, followed by a 11 × 1 convolution layer with 2 × 1 stride and 16 filters, another batch normalization layer, a ReLU layer, and a pool module. Next, the data were passed through a series of residual modules and pool modules, resulting in a 32 × 12 × 1024 array. A global pooling layer was followed by the last residual module. We divided it into 12 lead-specific feature maps with 1024 features. These feature maps were passed through a fully-connected layer with 1 neuron to generate the lead specific predictions. We designed an attention mechanism based on a hierarchical attention network to concatenate these blocks, increasing the interpretive power of DLM. The attention module was comprised of a fully connected layer with 8 neurons, followed by a batch normalization layer, a ReLU layer, and a fully-connected layer with 1 neuron to generate the weights of each lead. Attention scores were calculated for each ECG lead and then integrated for standardization by the last linear output layer. The standardized attention scores were used to weight the 12 ECG lead outputs by simple multiplication. The 12 weighted outputs were summed and passed through a predicted module to give the final prediction value.

To increase the nonlinear adaptability and reduce monotonously linear predicted functions of outputs, we used the category-wise label encoding technology to code the HbA1c concentration. The range of HbA1c concentration was defined from 4.0% to 10.0%. We designed a 20 sigmoid output by an interval of 0.3. For example, the minimal HbA1c concentration of less than 4.0% is coded as (0, 0, 0, 0, 0, 0, 0, 0, 0, 0, 0, 0, 0, 0, 0, 0, 0, 0, 0, 0), the HbA1c concentration of 6.5% is coded as (1, 1, 1, 1, 1, 1, 1, 1, 1, 0, 0, 0, 0, 0, 0, 0, 0, 0, 0, 0), the HbA1c concentration of 8.0% is coded as (1, 1, 1, 1, 1, 1, 1, 1, 1, 1, 1, 1, 1, 1, 0, 0, 0, 0, 0, 0), and so on. The loss function is cross-entropy in these sigmoid outputs, and our network was trained to minimize the cross entropy loss. The final prediction was the sum of these values multiplied by 0.3 plus 4.0. For example, a prediction vector given by DLM is (1, 1, 1, 1, 1, 1, 1, 1, 0.9, 0.8, 0.7, 0, 0, 0, 0, 0, 0, 0, 0), which corresponds to an ECG-HbA1c concentration of 7.12%.

An oversampling process was implemented to ensure that rare cases with extreme HbA1c values were adequately recognized, which was based on weights computed on the prevalence of 20 equidistant intervals in the development cohort. In our study, the distribution of HbA1c was not uniform, therefore the ECGs with rare values were copies of existing samples at random to increase the number of observations. This ideally gives us a sufficient number of samples to play with [38,39]. However, we explored multiple oversampling strategies to maximize the model’s performance because ECG was related to gender and age [32]. Figure 2B shows the summary of four training strategies. The first strategy was the oversampling process based on the reciprocals of prevalence of 20 equidistant intervals in each batch (no match). The second strategy was to ensure a balanced gender distribution in each batch (gender-match). The third strategy was to additionally consider the weight of age, which was also computed on the prevalence of 20 equidistant intervals in the development cohort (age-match). The fourth strategy was matching both gender and age (gender/age-match). We compared the matching effects of the 4 trained DLMs using a full-scale development cohort. A sensitivity analysis using only ECGs from OPD (subset-1) and ECGs with corresponding HbA1c (subset-3) was conducted.

We trained these DLMs with a 32 batch size and used an initial learning rate of 0.001 using an Adam optimizer with standard parameters (β_1_ = 0.9 and β_2_ = 0.999). The learning rate was decayed by a factor of 10 each time the loss of the validation cohort plateaued after an epoch. To prevent the networks from overfitting, early stopping was performed by saving the network after every epoch and choosing the saved DLMs with the lowest loss on the validation cohort. The only regularization method for avoiding overfitting was the L2 regularization with a coefficient of 10^−4^ in this study.

Abbreviations: OPD, outpatient department; HEC, health examination center; IPD, in-patient department; EMR, emergency room; BMI, body mass index; SBP, systolic blood pressure; DBP, diastolic blood pressure; DM, diabetes mellitus; HTN, hypertension; HLP, hyperlipidemia; CKD, chronic kidney disease; STK, stroke, CAD, coronary artery disease; HF, heart failure; AF, atrial fibrillation; COPD, chronic obstructive pulmonary disease; HbA1c, glycated hemoglobin; GLU, glucose AC; eGFR, estimated glomerular filtration rate; BUN, blood urea nitrogen; Na, sodium; K, potassium; Cl, chloride; Ca, total calcium; Mg, magnesium; Alb, albumin; CRP, C-reactive protein; WBC, white blood cell count; PLT, platelet; Hb: hemoglobin; AST, aspartate aminotransferase; ALT, alanine aminotransferase; TG, triglyceride; TC, total cholesterol; LDL, low density lipoprotein cholesterol; HDL, high density lipoprotein cholesterol.

### 2.4. Statistical Analysis and Model Performance Assessment

Patient characteristics are presented as means and standard deviations, numbers of patients, or percentages where appropriate and were compared using either analysis of variance, Student’s t-test, or Chi-square test, as appropriate. All statistical analyses were completed in R version 3.4.4. The significance level was set as *p* < 0.05. We provided a series of DLMs with training via different strategies, and the optimal DLM was selected based on the highest AUC for detecting DM in the validation cohort. Moreover, the results of XGB model and elastic net were presented, which provided corresponding variable important rankings to explore the relationship between explainable features and HbA1c.

The primary analysis was to explore the diagnostic value on DM and severe DM in the follow-up cohort. The AUC, sensitivity (recall), specificity, precision, and F-measure are presented. Moreover, confusion scatter plots with mean absolute error (MAE) were used to compare actual HbA1c and ECG-HbA1c. The stratified analysis was also conducted. The secondary analysis was to explain the estimation residual between laboratory-based and ECG-based HbA1c. We explored the difference in characteristics in each ECG-HbA1c group sharing the same Lab-HbA1c. Linear regression or logistic regression was used for statistical testing where appropriate. Finally, we used univariable and multivariable Cox proportional hazard models to analyze the relationship between baseline characteristics and outcomes of interest. Hazard ratios (HRs) and 95% conference intervals (95% CIs) were used for comparison. A series of integration models were evaluated using the C-index as global performance to explore the additional contributions of ECG-HbA1c.

## 3. Results

Table 1 shows patient characteristics in the development, validation, and follow-up cohorts. Almost all characteristics were different among these three cohorts, which were grouped by date. This might reduce the generalizability of DLM if it was learned via spurious relationships. The number of mortalities was 61 (1.9%) during a median follow-up period of 4.5 years, and the incidence of new-onset CKD and HF was 8.3% (201) and 2.8% (86), respectively.

**Figure 3:**
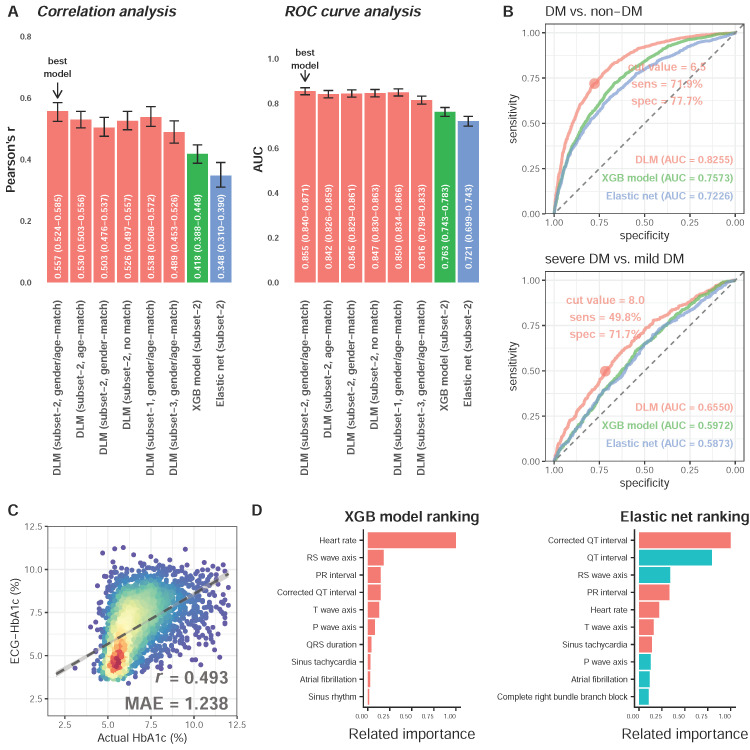
Analysis of deep learning model and traditional machine learning models. (**A**) The performance comparison of deep learning model (DLM) trained by 6 different weighting strategies in the validation cohort. The DLM (…) were made by predictions of deep learning models using different strategies. The XGB model and Elastic net demonstrated the corresponding predictions. The gender/age-matching strategy provides the highest correlation between estimated HbA1c and actual HbA1c. (**B**) Performance comparison for detecting DM and severe DM in the follow-up cohort. ROC curves were created from predictions of the deep learning model trained using a gender/age-matching strategy. Moreover, the performance of XGB model and elastic net were also presented. (**C**) Scatter plot between DLM predictions and actual HbA1c in the follow-up cohort. The *x*-axis indicates the true HbA1c from laboratory tests. The *y*-axis presents the predicted HbA1c from the deep learning model trained using a gender/age-matching strategy. Red points represent the highest density, followed by yellow, green, light blue, and dark blue. (**D**) Related feature importance ranking in XGB model (information gain) and elastic net (standard coefficient). There are only the top 10 important variables in each model, and the blue color demonstrates the negative relationship between variables and actual HbA1c.

We next explored a suitable DLM training strategy for subsequent analysis. Figure 3A shows that the HbA1c predicted by DLM with gender/age-match provided the highest AUC of 0.855 (95% CI: 0.840–0.871) for detecting DM, which was the most highly correlated with laboratory-based HbA1c (*r* = 0.557, 95% CI: 0.531–0.582). Figure 3B shows the performances of the best DLM, XGB model, and elastic net for detecting DM and severe DM in the follow-up cohort. The AUCs of DLM with gender/age-match, XGB model, and elastic net on DM was 0.8255, 0.7573, and 0.7226 in the follow-up cohort, respectively. Our ECG-HbA1c shows a sensitivity of 71.9% and specificity of 77.7% in the detection of DM. For patients with DM, we observed an AUC of 0.6550 using DLM for detecting severe DM in the follow-up cohort, which was better than the XGB model (0.5961) and elastic net (0.5884). Therefore, the ECG-HbA1c was defined as the estimation result of DLM with a gender/age-match. The scatter plot with Lab-HbA1c versus ECG-HbA1c is presented in Figure 3C. The mean absolute errors of Lab-HbA1c and ECG-HbA1c in the follow-up cohort was 1.238 with correlations of 0.493. Figure 3D shows the most important role of heart rate in the prediction of HbA1c in the XGB model, while a corrected QT interval, QT interval, followed by an RS wave axis played vital roles in the elastic net.

**Figure 4:**
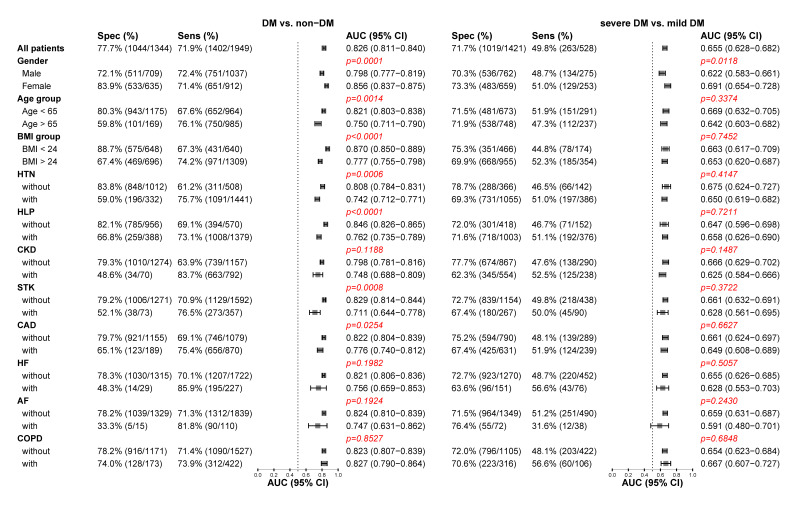
Stratified analysis for detecting DM and severe DM in the follow-up cohort. The DLM’s sensitivity and specificity to detect DM and severe DM are tabulated across a series of stratified analyses. The *p*-value was the significant test of strength of association, and a significance level was 0.0045 based on the Bonferroni correction.

Figure 4 shows that DLM performance was strong across all conditions to detect DM. The strengths of association, albeit widely inconsistent in different conditions for DM detection, were much higher for female and younger patients with fewer co-morbidities (HTN, HLP, and STK) and low BMI. The DLM exhibits higher AUCs with higher specificities in health patients, which indicated that patients with complex co-morbidities were more likely to be recognized as DM by ECG-HbA1c. This implied that patients with normal ECG-HbA1c but abnormal Lab-HbA1c (false negative) were young and healthy, while patients with abnormal ECG-HbA1c but normal Lab-HbA1c (false positive) were elderly with co-morbidities. Intriguingly, these patient characteristics had no impacts on the performance difference of severe DM detection.

**Figure 5:**
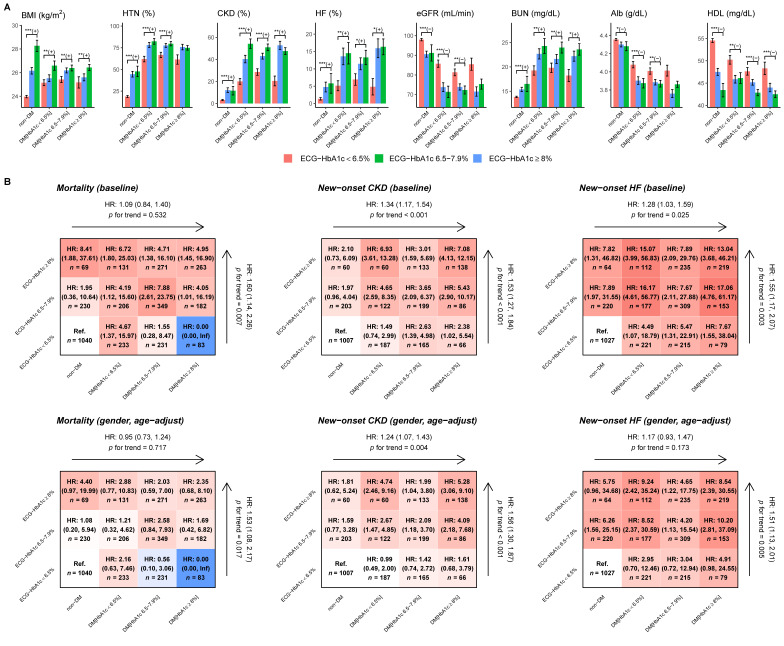
Characteristics and risk analysis in patients with actual HbA1c and ECG-HbA1c. (**A**) Patient characteristics in different ECG-HbA1c groups and real HbA1c groups. Bars represent the mean or proportion where appropriate and corresponding 95% conference intervals, which are adjusted by real HbA1c in each group via linear or logistic regression. Significant tests are based on the trend test (*: *p* for trend < 0.05; **: *p* for trend <0.01; ***: *p* for trend <0.001), and the sign represents the correlation direction. (**B**) Risk matrixes of ECG-HbA1c and HbA1c groups on DM related complications. The hazard ratios (HRs) are based on a Cox proportional hazard model before and after adjusting by gender and age. The color gradient represents the risk of corresponding group.

Figure 5A shows that the higher ECG-HbA1c groups exhibit higher BMI, higher prevalence of CKD/HF/HTN, worse kidney function (eGFR and blood urea nitrogen), lower Alb, and lower high-density lipoprotein cholesterol compared with the lower ECG-HbA1c groups, which are the risk factors for DM-related complications. Figure 5B shows outcome analysis of both DM/Lab-HbA1c and ECG-HbA1c. The false positive detection by DLM (ECG-HbA1c ≥ 6.5%) shows higher HRs on three outcomes of interest compared to the true negative (ECG-HbA1c < 6.5%) in patients without DM. Moreover, the false negative group (ECG-HbA1c < 6.5%) presented the lower risk of these outcomes compared to true positives (ECG-HbA1c ≥ 6.5%) in patients with DM. The dose response effects of ECG-HbA1c were significant on mortality (HR: 1.53, 95% CI: 1.08–2.17), new-onset CKD (HR: 1.56, 95% CI: 1.30–1.87), and new-onset HF (HR: 1.51, 95% CI: 1.13–2.01) after gender and age adjustments, which was higher than the effects of Lab-HbA1c (HR of mortality: 0.95, 95% CI: 0.73–1.24; HR of new-onset CKD: 1.24, 95% CI: 1.07–1.43; HR of new-onset HF: 1.17, 95% CI: 0.93–1.47). All the results demonstrated the beneficial role of ECG-HbA1c on the prediction of the cardiovascular disease outcomes compared to Lab-HbA1c.

**Figure 6:**
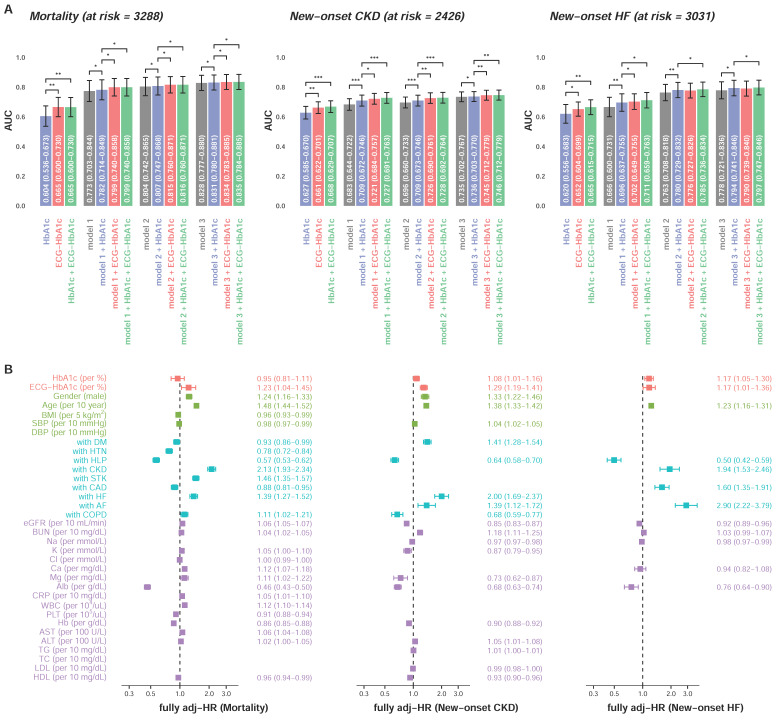
Additional contributions of ECG-HbA1c on DM related complications. (**A**) A Cox proportional hazard model and C-index are used as the performance assessment for a series of models. The model 1 includes significant demographic data, the model 2 includes variables in model 1 and additional significant disease histories, and the model 3 includes variables in model 2 and additional significant laboratory tests. Abbreviations: *, *p* < 0.05; **, *p* < 0.01; ***, *p* < 0.001. (**B**) The multivariable analyses of the models with best performance (model 3 + HbA1c + ECG-HbA1c) described above. The risk score can be calculated based on these coefficients to provide the corresponding C-index as above.

Figure 6A shows additive effects of ECG-HbA1c. In the mortality analysis, the ECG-HbA1c provided a C-index of 0.665 (95% CI: 0.600–0.730) which was significantly higher than Lab-HbA1c (C-index = 0.604, 95% CI: 0.536–0.673). After full adjustments, ECG-HbA1c provided significantly more information on mortality (C-index = 0.835 in model 3 + ECG-HbA1c) compared to Lab-HbA1c (C-index = 0.831 in model 3 + HbA1c), which is similar on the prediction of new-onset CKD. For the new-onset HF, the integration of Lab-HbA1c and ECG-HbA1c provided a higher C-index (0.665) compared to the Lab-HbA1c alone (0.620). In the full adjustment model including Lab-HbA1c, the integration of ECG-HbA1c significantly improved the model performance (C-index: 0.793 to 0.796, *p* < 0.05). Figure 6B shows the HRs of the full adjustment model with Lab-HbA1c and ECG- HbA1c on these three outcomes. The ECG-HbA1c independently provided risk predictions (HR of mortality: 1.23, 95% CI: 1.04–1.45; HR of new-onset CKD: 1.29, 95% CI: 1.18–1.41; HR of new-onset HF: 1.20, 95% CI: 1.03–1.39) in additional to a series of risk factors. Although the HRs of Lab-HbA1c were significant on the prediction of new-onset CKD (HR: 1.08, 95% CI: 1.01–1.16) and new-onset HF (HR: 1.17, 95% CI: 1.06–1.30), the effect of Lab-HbA1c was less than ECG-HbA1c. These results highlighted the strength of ECG-HbA1c to provide information on the unmeasured heart state.

## 4. Discussions

Our ECG-HbA1c provides an AUC of 0.8255 on DM screening in follow-up cohorts. The underlying characteristic differences in different ECG-HbA1c under the same Lab-HbA1c were analyzed, which revealed patients with higher ECG-HbA1c had more risk factors for DM progression. ECG-HbA1c provides additional information, although we had already adjusted for full baseline characteristics. We believe that ECG furnishes more information on latent cardiovascular factors compared to Lab-HbA1c, especially in unmeasured factors.

Several ECG manifestations have been proposed as a means of determining diabetic disease status. Diabetic rats exhibited prolonged ventricular depolarization time, decreased conduction velocity, and increased arrhythmia during reperfusion, which are reflected in ECG [40]. In human studies, increased resting heart rate [41] and longer atrial conduction time [42] were found to be correlated with DM. Long-term impaired fasting glucose was also observed to lead to accelerated RHR, ST-T changes, and arrhythmias in ECG [43]. Our data demonstrates that heart rate, corrected QT interval, QT interval, and RS wave axis were the most important ECG changes in the prediction of Lab-HbA1c during big data analysis. However, the detection of DM by ECG is difficult. DLM has been found to extract features unrecognizable to humans, such as obtaining cardiovascular risk factors from the retinal fundus [44], contributing to better performance than that of XGB models and elastic nets.

The advantage of DLM compared to traditional methods is to extract useful features automatically [20]. Recently, a study developed a DLM for screening DM via ECG with AUCs of 0.777 in an OPD experiment [45]. Through the larger database and augmentation from GLU, our DLM achieved an AUC of 0.8255. Moreover, both previous [45] and our own studies show that ECG based DM detection is more accurate for people with normal ranges of weight. The MAE of our noninvasive system (1.238) even approximately reached the 13 commercially available point-of-care HbA1c test devices ranging from −0.9 to 0.7 [46]. Importantly, our study further explores the meaning of predicting error, and finally points out the poor conditions in patients with higher ECG-HbA1c. ECG-HbA1c may be used to predict DM-related progression, which is critical in the identification of high-risk groups.

Although Lab-HbA1c may be the most important factor for prediction of DM progression [33], large amounts of missing data might preclude analysis by retrospective electronic medical records [16]. For example, cholesterol values were available for fewer than 30% of patients due to fewer measurements [47], necessitating a substitute, such as BMI, for the assessment of cardiovascular health [48,49]. Moreover, Lab-HbA1c might not be a perfect index for evaluating DM, especially in aged patients without DM [50,51]. Age-dependent HbA1c reference intervals for the diagnosis of DM have been proposed [52]. Our data demonstrated that ECG-HbA1c might be feasible when missing Lab-HbA1c values, and even has a higher predictive ability in regard to mortality, new-onset CKD and HF compared to Lab-HbA1c. Moreover, our data demonstrates that patients with higher ECG-HbA1c under the same laboratory-based HbA1c present increased risk factors for DM progression, indicating that ECG-HbA1c provides additional predictive information, even when the Lab-HbA1c is available. Similarly, it had been suggested that patients with higher ECG age under the same chronological age usually have a higher incidence of hypertension, CAD, or low ejection fractions [32]. Patients with abnormal ECG-based ejection fractions also exhibit a fourfold increased risk for developing future ventricular dysfunction [27]. Taken together, these results emphasize the beneficial effects of an ECG-based system for screening DM and predicting its progression, which warrants further validation in large-scale community studies.

The strength of our study is in conducting a series of experiments to apply different training strategies involving epidemiological perspectives and the gender/age-matching strategy with simulated HbA1c based on GLU to demonstrate the best performance. The matching strategy avoids the DLM learning spurious correlations, which maximizes additional notable ECG features. A previous study demonstrated the superiority of a matching strategy that avoids identifying discharge notes of neoplasms using negative terms, such as pregnancy [53]. Although matching strategies may not substantially increase DLM performance, they learn causality, which improves extrapolation. Gender and age are related to DM [54,55], and previous studies have shown correlations among gender, age, and ECG [32]. These relationships have led us to consider the possibility of confounding effects whenever these factors causally influence both ECG and HbA1c [56]. The gender/age-matching strategy not only provides a higher correlation, but also shows a lower correlation with age and gender. To the best of our knowledge, there is no DLM research that considers these potential confounding effects. Future medical DLM research may need to further analyze the source of predictive power and try to use matching strategies to improve learning quality.

Some limitations of this study should be acknowledged. First, this is a hospital-based retrospective study. Our data indicate the improved value of ECG-HbA1c, and we consider a community-based prospective study necessary to validate the effect of ECG-HbA1c. Second, ECG characteristics may vary by race, although the diagnostic performance of DLM may be still stable [57]. An international study involving different racial and ethnic groups should still be conducted to validate the advantage of ECG-HbA1c. Third, DM can be classified into 4 types, including type 1 diabetes, type 2 diabetes, gestational diabetes mellitus, and specific types of diabetes due to other causes [35]. Although type 2 diabetes is the most predominant type in the study, we could not provide the detailed type of diabetes of each patient. Finally, the “black box” of DLM necessitates our ECG-HbA1c being more transparent [58]. Although traditional explainable models reveal some clues, their performances are significantly worse than that of DLM. Further studies should explore the relationship between ECG morphological findings and DM severities.

## 5. Conclusions

In this study, we developed a novel biomarker, ECG-HbA1c, for predicting the risks and progression of DM and its related complications. In addition to clinical practice, our study creates a new avenue for using matching strategies for training DLMs, which avoids learning spurious correlations. Moreover, ECG is a simple, inexpensive, and noninvasive test that is suitable for applications in large-scale community settings. ECG-HbA1c is not only considered as a tool for initial DM screening, but also provides additional information on DM progression, even with available laboratory data. Although further studies are necessary, this system provides promising ECG-based indicators to promote health care quality in patients with DM.

## Figures and Tables

**Table 1:** Patient characteristics and laboratory results in development, validation, and follow-up cohorts.

	Development Cohort (*N*/*n* = 57,185/104,823)	Validation Cohort (*N*/*n* = 1539/2190)	Follow-Up Cohort (*N*/*n* = 3293/3293)	*p*-Value
Location				<0.001
OPD/HEC	56,511 (53.9%)	2190 (100.0%)	3293 (100.0%)	
IPD/EMR	48,312 (46.1%)	0 (0.0%)	0 (0.0%)	
Gender (Male)	59182 (56.5%)	1124 (51.3%)	1746 (53.0%)	<0.001
Age (years)	60.9 ± 17.1	56.0 ± 14.8	58.8 ± 15.0	<0.001
BMI (kg/m^2^)	25.2 ± 6.0	24.8 ± 3.9	25.5 ± 4.2	<0.001
SBP (mmHg)	136.0 ± 27.9	130.3 ± 25.0	134.4 ± 26.7	<0.001
DBP (mmHg)	79.3 ± 17.1	79.3 ± 14.8	79.5 ± 15.4	0.752
Disease history				
DM	50,176 (47.9%)	984 (44.9%)	1949 (59.2%)	<0.001
HTN	42,116 (40.2%)	846 (38.6%)	1773 (53.8%)	<0.001
HLP	41,117 (39.2%)	880 (40.2%)	1767 (53.7%)	<0.001
CKD	34,246 (32.7%)	438 (20.0%)	862 (26.2%)	<0.001
STK	13,893 (13.3%)	216 (9.9%)	430 (13.1%)	<0.001
CAD	24,474 (23.3%)	508 (23.2%)	1059 (32.2%)	<0.001
HF	6693 (6.4%)	119 (5.4%)	256 (7.8%)	0.001
AF	4983 (4.8%)	70 (3.2%)	125 (3.8%)	<0.001
COPD	13,555 (12.9%)	239 (10.9%)	595 (18.1%)	<0.001
Laboratory test				
HbA1c (%)	7.0 ± 1.8	6.3 ± 1.4	6.6 ± 1.6	<0.001
GLU (mg/dL)	119.1 ± 49.3	115.5 ± 43.9	123.2 ± 49.1	<0.001
eGFR (mL/min)	81.6 ± 36.2	89.2 ± 27.1	84.5 ± 30.3	<0.001
BUN (mg/dL)	22.1 ± 19.6	16.5 ± 9.7	18.8 ± 13.7	<0.001
Na (mmol/L)	137.8 ± 4.8	139.0 ± 3.8	138.5 ± 4.2	<0.001
K (mmol/L)	4.0 ± 0.5	4.1 ± 0.4	4.1 ± 0.5	<0.001
Cl (mEq/L)	103.3 ± 5.0	103.8 ± 3.7	103.5 ± 4.4	<0.001
Ca (mg/dL)	9.0 ± 0.7	9.2 ± 0.5	9.1 ± 0.6	<0.001
Mg (meq/L)	2.1 ± 0.3	2.1 ± 0.2	2.1 ± 0.3	0.122
Alb (g/dL)	3.9 ± 0.7	4.2 ± 0.5	4.1 ± 0.5	<0.001
CRP (mg/L)	2.8 ± 5.5	1.4 ± 3.3	1.8 ± 3.9	<0.001
WBC (10^3^/uL)	8.3 ± 5.1	7.0 ± 4.7	7.4 ± 3.2	<0.001
PLT (10^3^/uL)	235.4 ± 81.3	237.3 ± 68.1	234.9 ± 71.7	0.504
Hb (mg/dL)	13.1 ± 2.3	13.6 ± 1.9	13.5 ± 2.1	<0.001
AST (U/L)	35.9 ± 119.8	22.3 ± 15.8	25.0 ± 21.8	<0.001
ALT (U/L)	31.8 ± 103.2	22.5 ± 17.0	25.0 ± 25.6	<0.001
TG (mg/dL)	136.6 ± 131.0	137.5 ± 104.7	145.7 ± 157.9	<0.001
TC (mg/dL)	172.0 ± 48.8	179.4 ± 38.3	178.5 ± 41.7	<0.001
LDL (mg/dL)	102.9 ± 37.5	108.2 ± 33.4	107.4 ± 34.9	<0.001
HDL (mg/dL)	46.7 ± 15.2	49.4 ± 13.6	48.5 ± 14.0	<0.001

*N* = number of patient; *n* = number of ECG.

## Data Availability

The data presented in this study are available on request from the corresponding author.
